# Resettlement Workforce Perspectives on Mental Health Care of Refugees

**DOI:** 10.3390/ijerph22081247

**Published:** 2025-08-09

**Authors:** Bibhuti K. Sar, Lesley M. Harris, Adrian J. Archuleta, Susan H. Rhema, Nicole B. Adams, Eva Nyerges, Doroty Sato

**Affiliations:** 1 Kent School of Social Work and Family Science, University of Louisville, Louisville, KY 40292, USA; lesley.harris@louisville.edu (L.M.H.); adrian.archuleta@louisville.edu (A.J.A.); susan.rhema@louisville.edu (S.H.R.); doroty.sato@louisville.edu (D.S.); 2 Special Education and Child Development, Cato College of Education, University of North Carolina at Charlotte, Charlotte, NC 28223, USA; nicoleadams@charlotte.edu; 3 Social Work Department, College of Social Work, University of Kentucky, Lexington, KY 40506, USA; eva.nyerges@uky.edu; 4 Department of Community Sustainability, College of Agriculture & Natural Sciences, Michigan State University, East Lansing, MI 48824, USA

**Keywords:** refugee mental health care, resettlement workforce, barriers, facilitators, refugee workforce perspectives, focus groups

## Abstract

Background: To identify the mental health care needs of resettled refugees, researchers have studied the perspectives of mental health service providers but have paid limited attention to the perspectives of individuals who work directly in resettlement agencies or in agencies that exclusively provide services to promote refugees’ self-sufficiency and integration—the refugee resettlement workforce—who routinely provide support, make referrals, and coordinate mental health care. To better inform programming and service delivery, this qualitative case study focuses on the perspectives of the resettlement workforce. Methods: Focus group interviews conducted with 48 refugee resettlement workforce members were analyzed for their perspectives on refugee mental health needs and care. Results: Thematic analysis revealed that their perspectives centered on barriers to (i.e., resettlement challenges, notions about mental illness, stigma associated with mental illness, inadequate access to mental health care, and limited technology literacy) and facilitators of (i.e., promoting mental health literacy, addressing stigma, providing specific and targeted training, mental health coordination, allies, and building programming capacity) refugee mental health care. A set of recommendations to minimize barriers and promote facilitators is presented. Conclusions: These findings corroborate previous research and inform the practices, programs, and policies that should be developed and implemented to support refugees’ mental health wellbeing, self-sufficiency, and community integration post-resettlement.

## 1. Introduction

Many of the 43.7 million refugees of the estimated 122.6 million forcibly displaced people worldwide [1] are subjected to political/religious persecution, war, violence, torture, assault, rape, separation from family, and loss of home and property prior to fleeing their homeland (pre-flight). And, while fleeing (in-flight), many endure physical and psychological harm, exploitation, and abuse. In resettlement (post-flight), they deal with resettlement and acculturation stressors as they work towards achieving self-sufficiency in a short period of time, which involves learning a new language, securing employment, handling all household expenses (i.e., rent, food, utilities), and integrating into their new community. Refugees demonstrate remarkable adaptability and resilience in managing and overcoming these adversities and challenges [2,3]. Some refugees, however, suffer from some degree of post-traumatic symptomology and mental health distress (i.e., feeling sad/depressed, anxious, helpless, hopeless) [4,5]. These reactions to painful and traumatizing circumstances, if unchecked, can be a risk factor for developing lasting physical, emotional, social, and psychological sequelae that exacerbate health and mental health issues, which can result in diagnostic-level psychiatric disorders (i.e., post-traumatic disorder), impeding successful resettlement [6,7,8,9]. One analysis, for example, found that the prevalence rates were 13% for diagnosed anxiety, 30% for depression, and 29% for PTSD. They were even higher for refugees’ self-reported anxiety (42%), depression (40%), and PTSD (37%). These rates were much greater than those for non-refugee populations across the world and people living in war or conflict zones [10]. It is important to note here that the accuracy and validity of these and other reported prevalence rates are questioned by practitioners and scholars. Practitioners have argued that refugees should not be diagnosed with mental illness for “normal reactions to intense human suffering” (p. E98) [11] and have indicated that such pathologizing and labeling is detrimental to refugees’ wellbeing and the helping process [11]. Scholars note that, in research, clear distinctions are not made between symptoms resulting from experiencing/being exposed to trauma and a diagnosable mental health condition, due in part to lack of accounting for cultural variations in the expression of traumatic stress symptoms and the use of measures that were not developed and tested using a refugee sample [12].

Within this context, researchers have studied the mental health care needs of refugees, primarily from the perspectives of mental health service providers. However, the perspectives of another key stakeholder, namely the refugee resettlement workforce have received limited attention despite being in the best position to illuminate the needs of those [refugees] they serve [13]. The refugee resettlement workforce is of all the individuals who work directly in resettlement agencies or in agencies that exclusively provide services to refugees to promote refugees’ self-sufficiency and integration. They may provide cash and medical assistance, language classes, employment and job readiness training and services; set up microenterprise and agricultural development and partnerships; and manage, coordinate, link and/or directly provide refugees with health, mental health, social, educational, legal and other needed services. Its members (a) routinely encounter refugees experiencing mental health distress and illness, and (b) make referrals and coordinate refugees’ access and use of mental health care. Hence, in this paper, their perspectives are described to more accurately inform the policies, programs, and practices implemented to support refugees’ resettlement, self-sufficiency, and community integration.

## 2. Literature Review

Perhaps because refugees’ ability to access and receive mental health care is shaped by the experiences and attitudes of health professionals [14,15], most of what is known about provider perspectives on refugees’ mental health care primarily are from studies involving service providers working external to the refugee resettlement workforce, such as mental health and primary care providers [16,17], local health care practitioners, traditional/spiritual healers, and humanitarian relief workers [17]; health and social service workers from clinics, hospitals, and mental health treatment facilities [13]; case managers, Muslim faith leaders, counselors/licensed social workers and health care providers [18]; and Directors/CEOs, managers, advisors, psychotherapists, mental health counselors/advocates, and art therapists [19].

The perspectives shared by mental health and other providers in these studies are that refugees are less likely to seek mental health services because of the stigma they perceive and/or their experiences associated with mental illness [13,18], resulting in unnecessary suffering. This is worsened by language barriers, the limited use of services due to cost [13,14,17,18,20], poor or inadequate provider communication about mental health diagnosis and treatment [16], and inadequate provider knowledge, preparedness, and practical training for properly conducting assessment and treatment, including addressing specific mental health conditions [14,17,18,20,21]. Providers also indicate that they frequently lack the manpower to access the needed available resources [17,18,20,21] to provide adequate mental health care. Lastly, they mentioned the weight of managing their own emotional strain and burnout as factors that negatively contribute [21] to their ability to provide the best possible mental health care to refugees.

In order to alleviate the barriers noted above, mental health and other providers have suggested leveraging and de-stigmatizing community-based mental health services [18] and offering training on cultural models of mental illness to providers to reduce the culturally specific barriers contributing to refugees’ underutilization of mental health services [14]. Furthermore, they advocated for cultural awareness training for providers, particularly those treating sexual minority refugees [22] with the belief that those who are introspective about their own cultural biases about gender and sexual orientation are in the best position to provide services to that population [23]. Finally, providers recommended focusing on key social determinants to improve community integration, quality of life, and mental health for asylum seekers and refugees [19].

Turning to studies exclusively focused on the perspectives of the resettlement workforce, Salami and colleagues [20] in Alberta, Canada, gathered the perceptions of fifty-three service providers on access to and use of mental health services by refugees and immigrants. Language, cultural interpretations of mental health, stigma around mental illness, and fear of negative repercussions if mental health issues were revealed are barriers to accessing mental health care. They recommended developing community-based services, addressing financial barriers, providing mental health training, enhancing collaboration across sectors in mental health service delivery, and expanding the role of interpreters and cultural brokers to overcome barriers to accessing mental health care. The research undertaken here seeks to corroborate these findings and add recommendations to reduce the discrepancy between the high prevalence of mental illness and the lower utilization of mental health services among resettled refugees and, as mentioned earlier, inform policies, programs, and practices supporting refugees’ resettlement, self-sufficiency, and community integration.

## 3. Materials and Methods

### 3.1. Informed Consent

The study discussed here was part of a broader needs assessment, which was approved by the first author’s university IRB (# 22.0669) (all members of the research team are or were at the first author’s university when the research was conducted) at the beginning of October 2022. As part of the consent process, a member of the research team reviewed the study procedures, as outlined in the preamble of the informed consent, with the recruited participants and screened them to ensure that they met the study’s eligibility criteria; this included those who were 18 years or older, could speak, read, and write in English, and were a current refugee services provider.

### 3.2. Setting

The setting for this study was a mid-South state, which ranks fourth per capita in refugee arrivals (325 refugee arrivals per 100,000 state population) in the United States. Refugees resettled are from countries with ongoing significant social, political, and/or economic strife, including but not limited to the Democratic Republic of Congo, Burma/Myanmar, Burundi, Somalia, Cuba, Ukraine, Afghanistan, Syria, Eritrea, Iraq, and Bosnia. The major resettlement regions are in the state’s urban/metropolitan areas, with a recent increase in the number of resettlements taking place in rural/suburban areas of the state. Over the last 30 years, three major refugee resettlement agencies have resettled over 30,800 refugees throughout the state.

### 3.3. Recruitment

Information about this study was shared with refugee resettlement agencies and refugee-serving community organizations throughout the state. Purposive and snowball sampling strategies were used to recruit potential participants who were current refugee service providers or service supervisors/administrators. Potential participants, once identified, were also encouraged to refer other individuals who might be eligible for participation in the study.

### 3.4. Design

A *descriptive* case study research design, a type of case study describing a phenomenon and the real-life context in which it occurs, was employed to explore provider perspectives [24]. This approach “facilitates exploration of a phenomenon within its context using a variety of data sources” and ensures that an issue is examined through a “variety of lenses which allows for multiple facets of the phenomenon to be revealed and understood” (p. 544) [24].

### 3.5. Sample

Forty-eight participants completed a screening and demographic questionnaire that asked about their age, gender, race, ethnicity, education level, length of employment, current job title, type of work, and whether they had engaged with and/or interacted with refugees in their work.

### 3.6. Data Collection

A total of ten focus groups were conducted by members of the research team who were paired up (authors other than the 1st and 5th authors teamed up in pairs to conduct the focus groups. One paired team consisted of the 2nd (lead) and 6th author, a second team consisted of the 3rd (lead) and 7th author, and a third team consisted of the 4th (lead) and 7th author). One member served as the lead/moderator and asked the questions while the other assisted with following up with any clarifying questions. Each focus group consisted of four to six participants and lasted up to two hours. Responses were audio recorded and later transcribed for analysis. Examples of the guiding questions posed to the refugee resettlement workforce providers included:

- What are the mental health needs of refugees for whom you provide services?
- Who are the individuals that have the most persistent, pressing, or underserved mental health needs?
- What are the challenges or barriers you have encountered when working with different cultural groups?
- What are the pre-migration and post-resettlement stressors that contribute to newly arrived adult refugees’ emotional distress that you have witnessed/observed?
- What are the ways to build capacity within communities to address the mental health needs of refugee populations?
- What are the limitations associated with mental health care of refugees?
- What are the ways to assist clients to overcome limitations associated with accessing mental health care?

### 3.7. Data Analysis

#### 3.7.1. Quantitative Data Analysis

Descriptive statistics (frequency, mean, range) were performed on the collected demographic data using SPSS 26^®^ to describe the focus group participants.

#### 3.7.2. Qualitative Data Analysis

The six steps of thematic analysis—familiarization, generating codes, generating themes, reviewing themes, defining/naming themes, and creating the report (Braun & Clarke, 2021)—guided the analysis of the focus group interviews. An essentialist/realist frame was employed in conducting the data analysis; this consisted of implementing an inductive approach to identifying themes that were strongly linked to the data, data-driven coding, and identifying themes at the semantic level that involved description and interpretation, theorized in relation to previous research [25].

The focus group interviews were transcribed by a professional transcription service (REV.com), and all identifying information was removed prior to data analysis. Dedoose^®^ (Version 9.0.17, https://www.dedoose.com/, accessed on 1 June 2024)) was used as a web-based qualitative data analysis platform to facilitate data organization and coding. Guided by the six steps of thematic analysis, the focus group transcripts were read and re-read several times by each of the research team members while noting initial ideas about the data. Then, initial codes were generated and assigned by systematically going through the transcripts line by line from beginning to end. As more of the transcripts were reviewed, the initial codes generated were revised to reflect the inclusion of the additional perspectives into the analysis. Inter-rater reliability tests were performed with coded transcripts to ensure agreement among research team members. Where there was no agreement, each excerpt and associated code were discussed and adjudicated to arrive at consensus. Research team members kept memos related to analytical decisions, consulted with each other, and discussed the relationships among codes that emerged from the data. Next, the agreed upon sets of codes were grouped into similar categories and collated into potential themes. These themes were checked and reviewed for how well they reflected the codes and fit the original data from which the codes were developed. The themes were named and defined. Extracts from the data that reflected the developed themes were identified and were related back to the primary purpose of the research, which was to describe the perspectives of the refugee resettlement workforce.

## 4. Results

### 4.1. Sample Description

Forty-eight providers participated in the focus groups. They were primarily women, white, and, on average, were nearing 40 years of age (mean age: 39.06; SD = 11.826). Twenty-three (47.9%) self-identified as American, eight (16.7%) specified that they came from the African continent (i.e., Somalia, Democratic Republic of Congo), and two (4.2%) indicated that they had roots in the Middle East (i.e., Afghanistan, Lebanon), one (2.1%) in Europe (i.e., Germany), one (2.1%) in Asia (i.e., Myanmar), and one (2.1%) in Latin America (country not specified). Two (4.1%) indicated that they previously had a refugee status. Fifty percent (*n* = 24) of the participants were refugee resettlement workers, twenty-three percent (*n* = 11) were mental health care coordinators, fifteen percent (*n* = 7) were case manager/direct service workers (i.e., family coach, community health care workers, domestic violence coordinators), six percent (*n* = 3) were health care providers (i.e., nurse practitioner, community health care workers), and six percent (*n* = 3) were program directors. Almost all of them (89.5%, *n* = 44) had earned either a graduate (Master’s, JD, or PhD) or undergraduate (Bachelor’s or Associate’s) degree. Their length of employment at their respective agencies ranged from 3 months to 38 years, with an average of 7.59 (SD = 8.78) years (see Table 1).

### 4.2. Focus Group Findings

The thematic analysis revealed eleven major themes, five of which are categorized as barriers to mental health care, and the remaining six as facilitators of refugee mental health care. In the barriers category are (1) resettlement challenges, (2) notions about mental illness, (3) stigma associated with mental illness, (4) inadequate access to mental health care, and (5) lack of or limited technology literacy. In the facilitators category are (1) promoting mental health literacy, (2) addressing stigma, (3) providing specific and targeted training, (4) mental health coordination, (5) cultivating allies, and (6) building programming capacity. Each of these categories is described in more detail below, starting with barriers.

#### 4.2.1. Barriers

Resettlement Challenges. Participants indicated that the lingering pre-migration and ongoing post-resettlement challenges experienced by refugees were barriers to adequately addressing refugee mental health distress and needs. Resettled refugees face the daunting tasks of securing employment, paying for household expenses, and finding childcare, confounded by ongoing financial struggles, socioeconomic inequity, and difficulty accessing language or interpreter services. They experience changes in their role status and the loss of extended family and social system support. This is compounded at times by ongoing family tensions and conflict, which may have originated pre-flight or during flight, but is most visible post-flight (resettlement). Rightly, these basic needs become primary, and when they are unmet, refugees are not able to focus on their mental health issues and fully participate in services. The focus group participants mentioned that mental health providers find themselves providing case management and other services rather than therapy because clients do not have their basic needs met. These challenges were highlighted for newly arrived refugees, especially homebound women caregiving for their children, men who are not vocal about their needs, and persons with serious health conditions manifesting symptoms resulting from past traumatic experiences. They were mentioned as having the greatest need but being the least engaged in mental health services, as has been found in previous studies [26,27]. One participant stated the following:

*“Specifically, Afghan population and Ukrainian population have very recent traumas with the war and separating from families when they escaped Afghanistan or Ukraine, leaving behind and some not even knowing where their family is or if they’re alive”.*

Relatedly, providers pointed out that when contrasted to members of the host community, refugees view their life circumstances, socioeconomic status, and social standing as contributing factors to their mental health distress as well.

*“It’s one thing to be poor and struggling in a refugee camp where everyone around you is poor and struggling, and it is another thing to get to the United States and be poor and struggling and then see what [life in] the United States could be. And there is this difference there that I think is a big stressor for clients. And then when they have children that are going to school with other kids that are having these really different life experiences, I think there’s a lot of stress there.”*

Notions about mental health, distress, and illness. Participants noted that the worldview of some refugees does not align with Western notions of mental illness nor what to do when such problems arise. It is difficult for some refugees to understand mental illness as a possible explanation.

*“A lot of them never heard what therapy or mental health, that kind of stuff is before and just maybe their lack of knowledge and understanding of what it is, how the process goes and how it will help them I think is a big issue.”*

Some participants emphasized that mental illness is often viewed as the norm among some refugees since it is also displayed by their immediate and extended family members, friends, and community members. Similarly, symptoms are accepted as opposed to dealt with, as they are determined to not be any worse than past experiences of the symptoms, which are often addressed through cultural means. Additionally, participants indicated that literacy (i.e., reading and writing) is frequently an issue, so providing information in order to educate was challenging. Consequently, this hindered service-seeking and delivery, resulting in a reduction in the perceived need or urgency to seek treatment, as conveyed in the following statement.

*“And if everyone around you has the same symptoms, maybe you don’t see it as being a mental health issue. If everyone around you has trouble sleeping because they’re having flashbacks to these events that have happened, then it’s normalized in the community. And so maybe it doesn’t register as being a problem”.*

Stigma associated with mental illness. Providers indicated that stigma associated with mental illness was a factor, if not the major factor, underlying the barriers and challenges they faced in facilitating mental health care for refugees. Some cultures do not have specific words to name mental illness, categorize or refer to it. In other cultures, mental health and mental illness are dichotomous rather than on a continuum from mental illness to mental health. Mental illness is often equated with terms such as “crazy” and “mad”, so bringing it up is taken to imply dishonor and bringing shame to the individual and their family. As pointed out by providers,

*“No one wants to talk about mental health. It’s very stigmatized. It would be insulting. It was almost like it would be an insult to the client if we referred them to you”*

[to mental health provider].

*“But if you tell a person there’s a mental health problem, I see that something is going on here wrong, with you, then it’s telling a person you’re crazy or at least that’s what it is perceived like. You did something wrong, it’s your fault and you’re a horrible human being now”.*

Several cultural, community, and family forces come into play to suppress addressing mental health care while elevating the stigma attached to it.

*“And their parents are like, they [Parents] have that stigma so they’re like, ‘That’s not real. You do not have mental health. That’s not real. Just be okay. And I went through all this and so you will be okay’. And that’s a lot of times what ends up happening”.*

*“I think that there’s always that roadblock if you will, of understanding on their part of what mental health looks like in our country and the stigma that they have. Whether it’s because they view things from a religious standpoint based upon their faith, I know a lot of our demographics, they see that as a weakness, which obviously in America I think that’s still a stigma here too”.*

Inadequate access to mental health care. Participants suggested that mental health providers’ (who were not part of the refugee resettlement workforce) limited cross-cultural knowledge and treatment expertise impact their ability to provide the best culturally responsive mental health care. Additionally, not all mental health providers with the cultural knowledge and expertise accept the insurance payment method (i.e., Medicaid) that refugees are covered under, making access and timely appropriate referrals a challenge. As one participant observed,

*“The model of healthcare we have currently is not conducive to really meeting the needs of clients that truly have the risk factors [for developing mental health problems]. So, I think a lot of what our refugee population needs is they need to build that trust with you and you’re not going to do that in 15 min. So, if you can really sit down and take 45 min with an interpreter to really build that relationship, you’re going to do a lot more good for them. But it’s just sad that our healthcare system doesn’t allow that. It’s very task oriented and it’s about reimbursement and I mean I could go on but I won’t”.*

When refugees are able to receive mental health care, they often face some sort of language barrier, such as (1) not having an interpreter, (2) there being a lack of providers who are bilingual and/or willing to see a client who is not fluent in English, and (3) having interpreters who are not able to translate the mental health concern and lack training on how to convey mental health concerns (in a non-stigmatizing way), as they only have received medical interpreter training. Additionally, some mental health providers who are able to provide mental health care find themselves providing case management and other services rather than therapy because the client does not have their basic needs met. Without addressing basic needs, mental health therapists find that the client is not able to focus on their mental health issues and fully participate in treatment. This is compounded by the lack of other existing service delivery systems operating in a manner that is understanding of refugees’ lived experience (i.e., trauma).

Technology literacy. Participants identified technological literacy as a barrier to refugees accessing and using mental health services. This includes limited language abilities, age, limited or no technology literacy, a lack of internet access, and difficulties understanding how to use technology processes (i.e., telehealth) effectively. These struggles with technology were stated by focus group participants to be prevalent among older refugees, especially those with limited levels of education. Younger refugees were described as being more familiar with technology, but limited English proficiency restricted many from navigating the process.

*“Technological literacy is really low among especially our older clients. And we’re very, very quickly getting up into a digital world. So, when you only have one cell phone and it’s kind of a $30 Walmart phone, it’s really difficult for me to show you how to pay your water bill, how to pay your rent online, how to pay your electric bill online.”*

***“*** *I see another very underserved population as older people… So maybe 50, 55 and up, who don’t have the support to take them to places, to help them remember their appointments. And sometimes they’re alone, they literally don’t have anybody. And just staying connected with mental health services is it seems most challenging for that group. And they can’t do the technical aspects of Zoom, so they need to get into the offices, but they don’t. Just not able to on a consistent basis”.*

Participants also discussed that they could not provide mental health services over the internet because some refugees have little or no access to Wi-Fi or no equipment at home. Participants pointed out that providers need more support from their IT system or allowances from their agency’s internal policies to use various means of technology (i.e., WhatsApp) in communicating with refugees.

*“Yeah, I would say we attempted to do virtual groups, I would say in May, June of 2020 using WhatsApp based on the community feedback…But we learned that some people didn’t have Wi-Fi at home, and they were using their cellular data. For video conferencing that just didn’t work. It wouldn’t sustain it. So, it wasn’t a sustainable approach. It was, unfortunately, not everyone was able to participate. So, people felt left out and people would get dropped, and it would interrupt. So, I haven’t found a solution to navigating some of the technological barriers in the community”.*

#### 4.2.2. Facilitators

The participants stated that promoting mental health literacy, addressing stigma, providing specific and targeted training (i.e., on cultural competency, screening for mental health distress), mental health coordination, cultivating allies, and building programming capacity were key to facilitating mental health care for refugees.

Mental health literacy. Participants made a number of suggestions regarding mental health literacy, including normalizing symptoms and treatment through education to reduce the shame and stigma experienced and associated with the use of mental health services. This was exemplified by one participant who stated the following:

*“I just want to add on to the piece that sometimes is also helpful to provide information about common symptoms that could be an indicator for mental health need. Sometimes people may be experiencing those symptoms but may not necessarily know these are physical manifestations of specific mental health needs”.*

Some participants suggested using cultural brokers or mentors to fill in the gaps in refugees’ knowledge and understanding of mental illness and trauma created by not having the language (i.e., there is not a word for mental illness in the language spoken) to name and describe mental illness and trauma (i.e., there is not a word for mental illness in the language spoken), and enhance refugees’ understanding of the mental health system and resources available in the community. Participants also pointed out that non-mainstream mental health providers were in need of mental health literacy. These providers would need to bridge the gap in their understanding of how mental health issues are manifested within the various cultural groups they encounter and how different cultural groups express those manifestations.

Addressing stigma. Participants suggested that overcoming mental health/mental illness stigma or at least mitigating its influence is not insurmountable. They indicated that it should begin with education and awareness efforts directed at refugees and community leaders to correct misconceptions and mitigate the lack of factual knowledge about mental illness.

*“And I think that part of the problem all across the board, no matter what culture is, there’s this big stigma with mental health care and it’s getting better thankfully. But, just that, I think maybe us taking a bigger approach to working that more into our cultural orientations and things like that to educate them on services that are available that promote being mentally healthy”.*

It was noted that regardless of where they work, all providers would benefit from critical self-reflection on how mental health issues are brought up and discussed with refugee clients.

*“You have to build trust with these clients first, because they’re so distrusting of government, they’re already distrusting of our office. So, before you’re going to say, “Oh, I think you need to go see a therapist”, they’ve got to learn to know you a little bit and learn that you’re here to help”.*

To decrease stigmatizing effects and increase refugees’ receptivity to mental health care, participants indicated that all providers should also receive training on how to communicate issues and concerns about mental health and mental illness within a trauma framework, to not further inflame their clients and family members’ distress over mental health symptoms and treatments. Interpreters were explicitly identified as needing training and education. As explained by participants,

*“Because a lot of people that have come from places that they’ve endured trauma, it’s shame. And so here we are, “Oh, tell us your trauma”. So, then it’s like the interpreters also preconceived ideas. So, it would be really good for them to actually be trained so that when they’re interpreting, it’s like, “Oh, this is not for a negative thing. This is not to brand you as you are sick. This is to enhance your life journey. And not to diminish what you went through”, but to say, “You know what? You went through this and we are going to work together to let you incorporate it into your life”.*

*“But interpreters may need more training on how to translate when it comes to that, because they make people back up because of… It’s really hard to translate the terms when it comes to mental health. Very, very hard. Even somebody like me talking about it. But when you’re translating it’s hard to get that word. And when you get it, if you are not careful, you are going to just end that conversation. So, language translators, they need some more trainings. It is not because they don’t know the language. It’s just, it’s a complicated, it’s not medical translation, no more medical translation”.*

Training and preparedness. For fellow workforce members, participants identified training on the use of mental health screening, intervention strategies, and skills as necessary, as well as identifying and responding to refugees’ mental health concerns. The specific topics suggested included using mental health screening tools, motivational interviewing, trauma-informed care strategies, crisis management, mandatory reporting, and responding to non-crisis mental health issues. They indicated that providers would benefit from critical self-reflection on how mental health issues are brought up and discussed with refugee clients. They should receive training on the mental health perspectives held by refugees and communication approaches that expand refugees’ understanding of services to increase refugees’ receptivity to mental health care. Increased cultural competency training was identified as a significant need for all staff and agencies (i.e., resettlement staff, mental health care providers, health care providers, community-based service staff, and law enforcement) who interface with refugees. Workforce wellbeing training, focusing on stress management and work–life balance, was also identified as a need in order for the workforce to be best prepared to support refugee mental health.

*“As far as the service providers, we need to learn or understand who we are working with. There are some of us who are not culturally competent. They are not from that culture. So, these clients had mental health issues, but there was a way that they were behaving before they got here. So, we need to know, understand, what did they used to resort to when there were any issues like this? They may not call it mental health, but it was something that was happening to them, but they used to treat it in some way. There are some that resort to spirituality. There are some that are resort to other healings. We need to know what helps”.*

Training interpreters on increasing their awareness, knowledge, and skills to enhance their understanding of mental health and work with fellow refugees is needed.

*“But interpreters may need more training on how to translate… It’s really hard to translate the terms when it comes to mental health. Very, very hard. Even somebody like me talking about it. But when you’re translating it’s hard to get that word. And when you get it, if you are not careful, you are going to just end that conversation. So, language translators, they need some more trainings. It is not because they don’t know the language. It’s just, it’s a complicated, it’s not medical translation”.*

*“And there’s, I think, a lack of that for interpreters to have that training and to have a better understanding that in certain cultures how things are expressed and how things need to be communicated so that there’s no misconnection in important facts with our clients who are suffering”.*

*“When it comes to interpreters, getting the right interpreter will be helpful. Or by right interpreter, I mean some interpreters may interpret word by word the way the provider said it and if they may interpret based on how they understood. I hear sometimes interpreters interpreting what I didn’t say to the client. Then I have to repeat and say, ‘This is what I mean, can you interpret like this?’ It is based on how they understood.”*

They also spoke about the need for training for mental health providers who work in other settings (i.e., community mental health centers, schools, and hospitals) and serve refugees in order to increase their knowledge about the lived experience of various refugee cultural groups and their views on mental illness and receptivity to mental health services. This stemmed from observations of the interactions between these mental health providers and refugees during the provision of mental health services.

*“I think there’s a really great need [for training] because of the fact that this is unprecedented for many of our mental health services here. We’ve heard from several different therapists and providers that they just don’t understand the type of trauma that these individuals have been through firsthand. So, they don’t know how to address that in accordance with knowing the cultural background, how they feel about it and how they would normally handle things”.*

Training was identified as a need regarding the use of tools to screen for mental health distress. Persons tasked with screening were interested in any materials that could further their understanding and ability to administer the screening tool. Training occurred “on the job”, often by shadowing an experienced screener and learning their process through the direct observation of how questions are asked to best answer difficult or confusing screening questions. Screeners expressed the most interest in training that would help them navigate difficult items and in receiving examples that have been proven effective in helping refugees understand what is being asked. Additionally, screeners requested information about the experiences of different populations and how their responses may vary based on the nature of their traumatic experiences (e.g., primary versus secondary trauma survivors). For example, some were interested in knowing whether some groups are more likely to express physical symptoms rather than mental health symptoms.

*“That’s right. And to ask that having the competent people administer the instrument is crucial. There should be some minimum baseline of who qualifies to do to administer. Because when [Name of Agency] came to us, one of the issues on the table is nobody’s testing positive for the thing. And it wasn’t the instrument; it was the person administering the instrument”.*

Mental health coordination. Participants described ways that the coordination of mental health care can be improved or enhanced. One suggestion was to increase the overall capacity of agencies to handle the ever-increasing demand for mental health coordination. Recruiting and hiring practices need to change and be more specific and targeted to recruit and hire persons who are representative of the cultural groups being served and have a specific set of skills (i.e., language, brokering, knowledge of culture). An examination of current practices should be undertaken to assess the strengths and challenges of existing partnerships and coordination by examining what partnerships are working well, and what strategies can/could be implemented to strengthen existing partnerships. There is also a need to establish greater collaboration and exchanges of peer support among those working with refugees across disciplines. The strategies offered to accomplish this included training/cross-training and education about the mission, goals, and objectives of other agencies, and sharing knowledge about the lived experience of refugees who need and are seeking mental health care.

*“I think overall, the opportunity to share with regular collaboration among different providers as far as case workers and mental health providers and physical health providers and social workers, just so that we’re all aware of what the situation is, instead of trying to do things piecemeal”.*

Cultivating Allies. Allies are mental health providers and partners in the community that are versed in trauma-informed services and acceptable to various refugee communities. Overall, participants discussed how allies supplement access to different types of care that are either no longer available to refugees, are not provided to refugees (i.e., not covered by insurance), or are associated with of an existing service gap in the community in medical, mental health, legal, or educational domains. The services provided by most allies are related to medical and mental health needs, where resettlement service providers need to identify someone with specialized skills to address a specific need. Participants discussed the need to leverage the resources of allies together (e.g., working with churches and medical providers to conduct a health fair) to meet the needs of this population, with churches, and schools providing critical linkages of support and access to refugees. Allies are willing to share their knowledge and experience and thus add to the community’s capacity in different service areas that lack the specialized knowledge to work with culturally diverse people.

*“Yes, I mean we have really good employers. That’s a good point. We have a lot of factories around here that have really taken on a large part of our refugees and have employed them and they’ve hired interpreters to help that process be smoother. So that would possibly be a resource that we could utilize some of those interpreters at their jobs”.*

*“There are not mental health providers in the resettlement agencies, or in the agencies that are doing the most direct services with these populations. And so, everything is referring out, and that there are some providers that we can connect with who are very culturally competent and work primarily with refugee or immigrant communities. And so, those few spaces, I think, are really good”.*

Participants emphasized reaching out to specific individuals within cultural communities and providing training to enhance their mental health knowledge and skills to work with fellow refugees, including conducting mental health awareness campaigns.

*“We have such a wealth of knowledge and experience in our communities already, but not the official license or whatever it may be to do the services. Funding could go towards recruiting people from those cultures. I know a number of people who informally do therapeutic services, but they’re like, ‘I don’t have the license so I can’t work at a practice.’ And it’s like, is funding the issue? Or are there other things? But yeah, I think there could be more space created for more.”*

Building programming capacity. Building programming capacity is focused on fostering knowledge, skills, and resources to provide mental health care for refugees. Building programming capacity was articulated by participants in terms of ideas for programming.

*“I know [name redacted] had an idea last week about having a health fair where it doesn’t focus on going to a zip [health] clinic per se, but it focuses on mental health, focuses on addiction, abuse, everything. So, it takes away that stigmatization. So, it takes it away and they realize there’s other people out here that’s going through the situation, This is how I can deal with it”.*

*“So also helping those population to get to know someone from the health provider, like assigning a face to a particular practice and helping them to build that relationship, I think could also be one key thing that providers can do for the population they’re serving”.*

*“We teach them [refugees] to fish, not fish for them, but they need that team [providers] first to come in and help coordinate with all those things and get them [refugees] to understand what’s available to them and then they can kind of graduate from the program and move on. I think if that’s what you wanted to do; I think that would be the best option is basically a wraparound type of program”.*

*“The mental health [problems] is leading more to relying on addiction that’s not being treated as well”.*

*“And I wish there were more programs in place for extra assistance, like a driver’s education program for adults, or outside of the English language training classes and things like that, have more layers of orientation culturally, things like that. We are in the process of working on, but I know that all of that has such an impact on their mental health and how they deal with things. And I think a lot of them just give up and throw their hands up in the air because it’s just, they get tired of looking for resources”.*

*“I don’t think there’s, to my knowledge, it’s more they’re [refugees] just referred through whoever. I wish there was something that meets that gap in the middle where they’re [providers] going out and really making the effort to educate and to say, we [providers] really do understand. We [providers] want to be able to help you [refugees] and we know what you’ve been through. We [providers] know what you’ve [refugees] encountered, and approach it from, how can we [providers] help you [refugees] or maybe inviting them [refugees] to a focus group to really express where their needs are, instead of just looking at the chain reaction of the trauma once they come here that happens…”*

Examples of suggested programming include finding and allocating resources for new programs (i.e., arts-based programming), funding for interpreters and intensive mental health case management, leveraging resources with community partners (e.g., transportation support, childcare and meeting space), increasing volunteer capacity, and making education available for practitioner self-care, secondary trauma, and wellbeing. As one participant noted,

*“We’re doing secondary traumatic stress with the providers. That’s what we’re speaking on. And then what’s warning signs of that, how to overcome that with self-care. And then also really instilling the work environment as being not just on the providers, but the work environment also being responsible for not causing so much secondary stress.*

Participants encouraged partnerships with cultural communities in order to design and implement mental health care interventions.

*“And also providing the opportunity for people who are just generally in the community, it doesn’t have to be a person who specifically has a mental health diagnosis, but just having maybe focus groups, if people are available or interested, they could help everyone become a little bit more informed and educated about what mental health means, what’s helpful, how would they seek services if they were available. Just so that it’s geared more towards something that’s practical for them and it’s not so prescriptive based on the provider’s part, like, “Well, this is what they need.” [It is] Getting input from the people that you’re serving”.*

Participants described needing a central coordinating agency or entity that could provide a hub for existing information, resources, and referrals to serve as a resource for refugees and practitioners.

*“I’d love a center to open up, that works directly with immigrants and refugees, and all of their staff would be trained, and have expertise in this area. There are other cities that do it”.*

## 5. Discussion

The findings of this study should be understood within the context of its design and implementation. The recruitment approach, self-selection, and agreement to participate were factors that affected who decided to participate in the focus group. Since the inclusion criteria specified that participants be able to speak, read, and write English, some non-fluent English providers may have opted out, and their voices may not have been captured. Additionally, some participants may have been cautious about what they shared in the focus groups, as they did not want to jeopardize their employment (even though confidentiality and how it would be shared were discussed) or be at odds with other focus group members. The focus group questions may not have fully captured the full range of refugee mental health needs observed and dealt with by the participants. The analysis of the interviews did not include checking the accuracy of the information provided by the participants by having them review the transcripts of the interviews or the results to ensure that they resonated with their experiences (i.e., member checking). The focus groups attempted to capture the perspectives of the refugee workforce working in organizations located in a specific geographic area, namely a mid-South U.S. state, which needs to be taken into account when considering the transferability of findings to similar settings and their applications more broadly.

Notwithstanding these limiting aspects of the study design, in this study, the thematic analysis of the focus groups identified five barriers to (i.e., resettlement challenges, notions about mental illness, stigma associated with mental illness, inadequate access to mental health care, and limited technology literacy) and six facilitators of (i.e., promoting mental health literacy, addressing stigma, providing specific and targeted training, mental health coordination, cultivating allies, and building programming capacity) of refugee mental health care. The barriers identified speak to resettlement challenges—i.e., pressures of acculturation, financial hardship, unemployment, socioeconomic inequity, and family conflict; lack of affordability—cost and inability to pay (i.e., lack of insurance and/or acceptance of payment method by provider); lack of accessibility—lack of available bilingual service providers, interpreters with experience in using and translating mental health terminology in a meaningful way; and lack of knowledge—limited refugee resettlement workforce as well as traditional mental health providers’ lack of cross-cultural knowledge and treatment expertise—which impacts their ability to provide culturally responsive mental health care. A lack of mental health literacy and pervasive stigmatization are also examples of contributing barriers, leading to a reduction in refugees’ perceived need for treatment, their urgency to seek treatment, and their receptivity to mental health services. These findings are consistent with previously reported perspectives of mental health providers i.e., [13,14,17,18,20] and echo those reported by Dumke et al.’s (2024) more recent exhaustive scoping review of studies regarding barriers to accessing mental health care for refugees and asylum seekers in high-income countries [28].

The facilitators identified through the thematic analysis emphasize training focused on knowledge and skill acquisition. Specifically, participants requested increased cultural competency training in some form for all staff and organizations (i.e., resettlement staff, traditional mental health care providers, healthcare providers, community-based service staff, and law enforcement) who serve or interface with refugees. They expressed the need for training for traditional mental health providers that focuses on the gap in their knowledge of various refugee groups’ views of mental illness and receptivity to mental health services. They requested training for interpreters to concentrate on strengthening interpreters’ ability to communicate mental health issues in a trauma-informed manner. Relatedly, participants suggested training individuals to conduct community awareness campaigns within their cultural communities. These findings have also been reported in previous studies i.e., [14,17,18,20,21].

In contrast to previous studies reviewed here on provider perspectives, this study identified and reinforced the importance of technology literacy (barrier) and cultivating allies (facilitator) to the mental health care of refugees. Technology literacy is known to have an impact on resettlement overall. Researchers have found that resettled refugees have limited skills navigating websites and determining the credibility of online information [29], and a lack of technology literacy is one of the main barriers to scaling up digital health applications for immigrants and refugees [30]. Digital technologies can be leveraged to support refugees in learning, finding employment, and establishing social connections and networks [31].

Cultivating allies is an important ingredient to successful resettlement. In this study, participants indicated that cultivating allies from outside and within refugee communities can supplement existing mental health resources, which are often limited within formal mental health service systems. A specific suggestion was to seek out and nurture allies within refugee communities to participate in refugee mental health care. This was particularly emphasized when discussing the need to increase (i.e., staff), strengthen (i.e., interpreters), and expand the workforce (i.e., allies, volunteers, and cultural community members) to provide services to refugees. This is consistent with the notion that refugees are more than just service recipients; they bring assets, strengths, skills, and resources, which can support and improve program access and usage [32]. Allies within refugee communities could be part of a “task sharing approach”—the formalized redistribution of care and duties usually provided by mental health specialists to non-specialists through training and supervision [33,34,35], similar to the training and preparation of community health workers who serve as a bridge between the refugee community and service providers via outreach, consultation, support, case management, referral and linkages to needed services. It is believed that such an approach can result in increased access to mental health services and greater connectedness, receptivity, and acceptance of mental health care and services [36].

### Recommendations

Based on the focus group findings presented here and the best practices gleaned from the literature and the field, the following recommendations are offered. They call for responsive and progressive policies, programs, and practices that can be pursued and instituted to adequately combat barriers and promote facilitators to strengthen refugee mental health care. First, the field needs to continue to engage in primary prevention. At the very least, refugees should be offered mental health information and education during resettlement and newcomer orientation. By making this standard practice, it may increase openness to mental health care and combat mental health stigma later in the resettlement process.

Next, reimagine training. Service providers within and outside the refugee resettlement workforce should be provided with specific, skill-focused experiential training involving problem-solving, case studies, modeling, and shadowing in order to communicate and engage with refugees (who are not a monolithic group) on concerns about mental illness within a trauma-informed care framework. This could increase receptivity to mental health care while decreasing instances of refugees and their family members having a distressed response to mental health symptoms and treatments due to poor or awkward provider communication.

Cultivate allies and partnerships. This can take many forms, such as including refugees with lived experiences in the design of interventions and development of peer-to-peer services, and valuing and enhancing the extended family’s role in the mental health care of their loved ones [37]. Allies are a source of both formal and informal care that is either no longer available to refugees or is not provided to refugees because of a lack of available resources or funding. They are integral to increasing and strengthening community partnerships, which offer necessary adjunct resources that are crucial in reducing barriers and improving access to mental health care.

Strengthen policies, programs, and practices. Standards of practice (SOPs) and a set of procedures to follow when screening for mental health distress should be established. Qualified persons with a specific set of skills (i.e., language, brokering, knowledge of culture) who are from the cultural groups being served should be hired. Institutional policies and practices that limit refugees’ full participation in mental health care should be challenged. Providers should advocate, seek, and provide resources that empower refugees with the means to fully participate in services (e.g., providing translated materials, making interpreter services more visible), including opportunities for improving technology literacy. Providers should acknowledge the diversity between and within refugee cultural groups. Not all refugees are the same, have the same needs, or have equal access to existing resources. Therefore, funding should be dedicated to specific program needs (i.e., interpretation, intensive case management, self-care, and secondary stress) to close the gap between well-established and growing resettlement communities.

Finally, emphasis should be on providing good care and practical support to refugees so they are successful at navigating very complex and bureaucratic legal systems, accessing housing, applying for benefits, finding employment, seeking education and training opportunities, establishing connection with their faith community, contacting/reuniting with family and relatives, and integrating into the larger host community, all of which are vital to refugees’ resettlement and long-term wellbeing. Such provisions of care and support make it more likely that refugees will have their basic needs met, which in turn can result in a lesser need for mental health interventions.

## 6. Conclusions

This thematic analysis of the perspectives of a cross-section of the resettlement workforce revealed the barriers to (i.e., resettlement challenges, notions about mental illness, stigma associated with mental illness, inadequate access to mental health care, and limited technology literacy) and facilitators of (i.e., promoting mental health literacy, addressing stigma, providing specific and targeted training, mental health coordination, allies, and building programming capacity) refugee mental health care. These findings can inform the direction of future research as well as the development and implementation of practices, programs, and policies to support refugees’ mental health and wellbeing, self-sufficiency, and community integration post-resettlement.

## Figures and Tables

**Table 1:** Demographic characteristics of focus group participants (N = 48).

Characteristic		n	%
Gender	Female	33	67.3
	Male	14	28.6
	Not Stated	1	2.0
Race	White/Non-Hispanic	31	63.3
	Black/African American	12	24.5
	Hispanic	3	6.1
	Not Stated	2	4.1
Heritage	American	23	47.9
	African (from Africa)	8	16.7
	Middle Eastern	2	4.1
	Latin American (Latino)	1	2.1
	European	1	2.1
	Asian	1	2.1
	Not Clearly Specified/Stated	12	25.0
Highest Degree Earned	High School Graduate	1	2.0
	Bachelors	20	40.8
	Masters	20	40.8
	Doctorate	4	8.2
	Associate degree	1	2.0
	Not Stated	3	6.1
Role/Position	Refugee Resettlement Worker	24	50.0
	Mental Health Care Coordinator	11	23.0
	Direct Service/Case Management Provider	7	15
	Health Care Provider	3	6
	Program Director	3	6
Age	Mean: 39.06	SD= 11.826	Range=23 to 60 years
Length of Employment (N = 36)	Mean: 8.28 years	SD= 9.01	Range = 3 months to 38 years

## Data Availability

The data are not publicly available due to privacy, ethical, and IRB requirements.
